# Parvovirus B19-Induced Lower Motor Neuron Facial Nerve Palsy and Cutaneous Small-Vessel Vasculitis: A Case Report

**DOI:** 10.7759/cureus.69181

**Published:** 2024-09-11

**Authors:** Rishabh Rawat, Geeta Kampani, Kunal Saini, Ravi Talapa

**Affiliations:** 1 Internal Medicine, Atal Bihari Vajpayee Institute of Medical Sciences and Dr. Ram Manohar Lohia Hospital, Delhi, IND

**Keywords:** cutaneous small-vessel vasculitis, facial nerve palsy, immunosuppressive therapy, neurological manifestations, parvovirus b19

## Abstract

Parvovirus B19, commonly associated with erythema infectiosum in children, can also present with various clinical manifestations in adults, including arthropathy, myocarditis, vasculitis, and neurological complications. This case report describes a 39-year-old male who presented with fever, rash, and polyarthralgia, followed by the sudden onset of left-sided facial weakness and slurred speech. Clinical examination revealed lower motor neuron facial nerve palsy and signs consistent with cutaneous small-vessel vasculitis. Extensive laboratory investigations confirmed the presence of parvovirus B19 DNA, while tests for other common viral infections and autoimmune markers were negative.

The patient was treated with oral prednisolone, resulting in significant improvement in his symptoms over the course of one month. This case highlights the rare but important association between parvovirus B19 infection and both neurological and dermatological manifestations. It underscores the need for healthcare providers to consider viral etiologies in the differential diagnosis of facial nerve palsy and vasculitis, particularly when presented with concurrent symptoms of systemic infection. Early diagnosis and appropriate management are crucial for improving patient outcomes in such atypical presentations. This report adds to the growing body of literature on the diverse clinical manifestations of parvovirus B19, emphasizing the importance of recognizing and treating these rare complications promptly.

## Introduction

Parvovirus B19 is commonly known for causing erythema infectiosum in children but also presents with various clinical manifestations in adults, including arthropathy, myocarditis, vasculitis, and neurological complications such as encephalitis, meningitis, and peripheral neuropathy [1]. The association between parvovirus B19 and lower motor neuron (LMN) facial nerve palsy remains rare and scarcely documented in the medical literature [2].

Cutaneous small-vessel vasculitis is an immune-mediated inflammation of the small blood vessels in the skin that can be triggered by various factors, including infections, medications, and autoimmune and systemic diseases. The link between parvovirus B19 and cutaneous small-vessel vasculitis adds to the complex interplay between viral infections and autoimmune responses [3].

In this case report, we describe a unique clinical presentation of a patient with concurrent LMN facial nerve palsy and cutaneous small-vessel vasculitis attributed to parvovirus B19 infection. This case highlights the need for heightened clinical awareness and consideration of parvovirus B19 as a potential etiological agent in atypical presentations involving both neurological and dermatological manifestations.

## Case presentation

A 39-year-old male presented with complaints of fever, rash, and multiple joint pains for 25 days, along with an inability to close the left eye for two days. The fever was continuous, associated with chills for 25 days, undocumented, mainly occurring at night and temporarily relieved by antipyretics. It was associated with the appearance of rashes over the trunk and upper and lower limbs, including palms and soles. Initially, the rashes were non-itchy, reddish, and papular for 10 days, and then turned hyperpigmented blackish macules. They were associated with multiple joint pains in all four limbs of inflammatory type, which was associated with early morning stiffness.

The patient also complained of the inability to close the left eye for the last two days. It was sudden in onset, associated with drooling of saliva from the left angle of the mouth and slurring of speech. There was no history of sensory loss, weakness of any limb, ear discharge, or any comorbidities.

**Figure 1:**
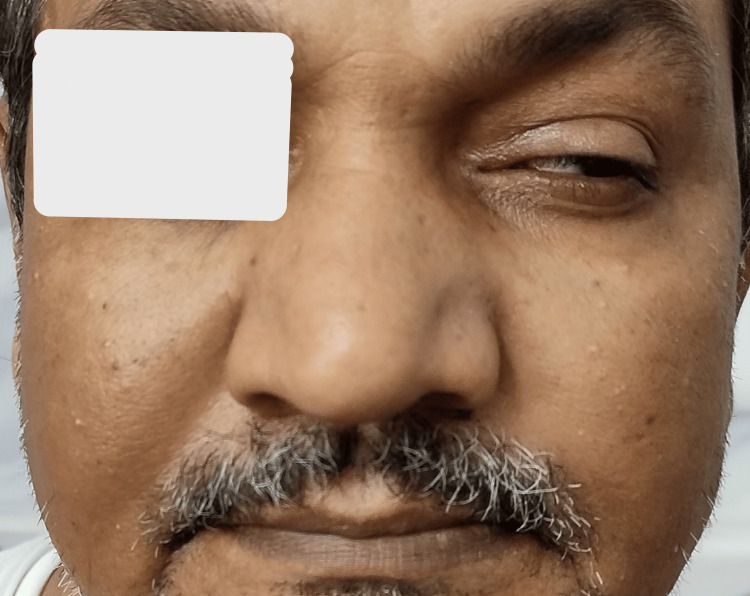
Patient with left-sided lower motor neuron facial palsy showing unilateral absence of nasolabial fold and incomplete eye closure.

During the general physical examination, the patient was conscious and oriented, with a blood pressure of 116/72 mmHg, pulse rate of 99 beats/minute, oxygen saturation of 98% on room air, and random blood sugar level of 116 mg/dL. Multiple healed hyperpigmented, non-blanchable papules were seen on the trunk, bilateral upper and lower limbs extensor aspect, palms, and soles suggestive of a resolved cutaneous small-vessel vasculitis. Bilateral wrists, elbows, and knees were tender and swollen. Neurological examination revealed isolated LMN type left VIIth nerve palsy, as shown in Figure 1. Other systemic examinations of the patient were normal.

The patient underwent a series of investigations summarized in Table 1. The combination of clinical findings, especially the presence of LMN facial nerve palsy, the history of a preceding fever with a rash, joint pains, and the healed skin lesions, led to a suspicion of a viral etiology. Parvovirus B19 infection was strongly considered due to its known association with similar systemic manifestations. The diagnostic workup confirmed the presence of parvovirus B19 DNA in the blood through real-time polymerase chain reaction (PCR), establishing the diagnosis.

**Table 1:** Laboratory and diagnostic test results of the case.

Parameter	Result	Normal reference range
Hemoglobin	12.6 g/dL	13.5–15.5 g/dL
Total leukocyte count	11,600/mm^3^	4,000–11,000/mm^3^
Platelet Count	220,000/mm^3^	150,000–450,000/mm^3^
Total Protein	6.7 g/dL	6.0–8.3 g/dL
Albumin	3.1 g/dL	3.5–5.0 g/dL
Globulin	3.6 g/dL	2.0–3.5 g/dL
Aspartate transaminase	88 U/L	10–40 U/L
Alanine transaminase	140 U/L	7–56 U/L
Alkaline phosphatase	128 U/L	44-147 U/L
Total bilirubin	0.9 mg/dL	0.1–1.2 mg/dL
Direct bilirubin	0.5 mg/dL	0–0.4 mg/dL
Sodium	139 mmol/L	135–145 mmol/L
Potassium	4.7 mmol/L	3.5–5.0 mmol/L
Calcium	8.8 mg/dL	8.5–10.2 mg/dL
Phosphate	4.8 mg/dL	2.5–4.5 mg/dL
Creatine kinase	21 U/L	38–174 U/L
Creatinine	1.0 mg/dL	0.6–1.2 mg/dL
Urea	44 mg/dL	7–20 mg/dL
Uric acid	7.3 mg/dL	3.5–7.2 mg/dL
Lactate dehydrogenase	431 U/L	140–280 U/L
Quantitative C-reactive protein	34.3 mg/dL	<1 mg/dL
Erythrocyte sedimentation rate	14 mm/hour	<20 mm/hour
Serology for malaria, dengue, leptospira	Negative	Negative
Serology for HIV, hepatitis B, hepatitis C	Negative	Negative
Urine routine	Normal	Normal
Anti-nuclear antigen, anti-neutrophil cytoplasmic antibodies, rheumatoid factor, anti-cyclic citrullinated peptide	Negative	Negative
Serum procalcitonin	Negative	Negative
Blood cultures	Negative	Negative
Herpes simplex virus DNA, varicella-zoster virus DNA, Epstein-Barr virus DNA, cytomegalovirus DNA	Not detected	Not detected
Skin lesion biopsy	Not done (healed lesions)	Not applicable
Parvovirus B19 DNA (real-time polymerase chain reaction)	Detected	Not detected

The patient was advised oral prednisolone 60 mg once a day for seven days, followed by tapering doses over the next two weeks along with supportive measures such as carboxymethyl cellulose eye drops, eye patching at night, and oral paracetamol 500 mg SOS. Upon follow-up after one month, the patient’s symptoms had improved.

## Discussion

Parvovirus B19 is recognized for its hematological effects and its association with arthropathy, but its neurological and vasculitic complications are increasingly documented. The case described here, involving LMN facial nerve palsy and cutaneous small-vessel vasculitis, adds to the limited but growing body of literature associating parvovirus B19 with diverse clinical manifestations.

Neurologic and vasculitic manifestations

The neurological complications of parvovirus B19, although uncommon, are significant. Facial nerve palsy, particularly the LMN type, is a rare manifestation. In a systematic review, Barah et al. identified various neurological aspects associated with parvovirus B19, including encephalitis, meningitis, and peripheral neuropathy, highlighting the need to consider this virus in the differential diagnoses of neurological conditions [2]. Similarly, Watanabe and Kawashima reported that acute encephalitis and encephalopathy are common neurological manifestations in children with parvovirus B19 infection [4]. Fukuta et al. reported one case involving a 19-month-old boy with facial nerve palsy diagnosed via PCR and serology, highlighting the potential for parvovirus B19 to affect the facial nerve and suggesting that it should be considered in the differential diagnosis of LMN facial palsy [5]. Another review of 81 cases of neurological disease linked to parvovirus B19 by Douvoyiannis et al. reported that while encephalitis and meningitis were more common, peripheral nervous system involvement, including facial nerve palsy, was also observed, particularly in immunocompetent individuals [6].

Parvovirus B19 is also known to cause various forms of vasculitis. Kerr discussed the virus’s role in triggering autoimmune responses that can lead to vasculitic processes, such as leukocytoclastic vasculitis and Henoch-Schönlein purpura, by demonstrating the direct localization of viral DNA to endothelial cells [7]. Ferreira et al. further illustrated the virus’s capacity to trigger vasculitis, reporting a case of encephalopathy associated with parvovirus B19 and *Haemophilus influenzae* meningitis in a newborn, highlighting the complex interplay between viral infections and autoimmune responses [8]. In this case report, we describe a unique clinical presentation of a patient with concurrent LMN facial nerve palsy and cutaneous small-vessel vasculitis attributed to parvovirus B19 infection.

Immune-mediated mechanisms

The pathogenesis of parvovirus B19-induced autoimmune phenomena such as vasculitis and facial nerve palsy is complex and multifactorial. Molecular mimicry, where viral proteins resemble host proteins, may induce autoimmunity. Parvovirus B19 infection has been implicated in the production of autoantibodies which is a key factor contributing to various autoimmune diseases, including systemic lupus erythematosus and rheumatoid arthritis [7]. Valle et al. also reported on the virus’s potential to trigger autoimmune encephalitis, further supporting the role of immune-mediated mechanisms in the pathogenesis of parvovirus B19-related complications [9].

Diagnostic approaches

Clinicians must select the appropriate test to ensure accurate detection of parvovirus B19 infection based on the patientHaemophilus influenzaes immune status. In immunocompetent patients, serological testing is recommended for detecting parvovirus IgM antibodies indicative of recent infection. However, in immunocompromised patients, who may not mount an adequate antibody response, molecular assays such as PCR that detect viral DNA are more reliable [10].

Therapeutic implications

The guidelines for treatment of LMN seventh nerve palsy recommend early administration of oral steroids within 72 hours of symptom onset which may be combined with antiviral therapy [11]. Corticosteroids are especially effective in viral-induced autoimmune conditions such as parvovirus B19-associated facial nerve palsy and cutaneous small-vessel vasculitis [12]. In our case, oral prednisolone led to the resolution of symptoms.

In more severe or refractory cases, intravenous immunoglobulin (IVIG) is considered, particularly for chronic anemia, myocarditis, or central nervous system vasculitis caused by parvovirus. IVIG helps modulate the immune system and neutralize autoantibodies. Other immunosuppressants, such as azathioprine, may be used for persistent cases but require careful monitoring to avoid excessive immunosuppression.

Timely intervention with appropriate therapies is critical for favorable outcomes, as demonstrated by the significant improvement in this patient’s condition following corticosteroid treatment. Long-term follow-up is essential to monitor for relapses. While relapses of parvovirus B19-induced facial palsy are rare, related conditions such as parvovirus B19-associated pure red cell aplasia have shown relapse rates as high as 33.9%, particularly in immunocompromised individuals undergoing IVIG therapy [13].

## Conclusions

This case report underscores the importance of considering parvovirus B19 in the differential diagnosis of atypical neurological and vasculitic presentations, such as LMN facial nerve palsy and cutaneous small-vessel vasculitis. The successful management of the patient’s symptoms with immunosuppressive therapy highlights the potential for effective treatment when parvovirus B19 is identified as the underlying cause. This case also emphasizes the need for clinical awareness of the diverse and sometimes rare manifestations of parvovirus B19. Ongoing research and case documentation are essential to better understand and manage the broad spectrum of complications associated with this virus.
